# Thromboelastography in acute immunologic reactions: a prospective pilot study

**DOI:** 10.1016/j.rpth.2024.102425

**Published:** 2024-04-27

**Authors:** Calvin Lukas Kienbacher, Christian Schoergenhofer, Gerhard Ruzicka, Jürgen Grafeneder, Christine Hufnagl, Bernd Jilma, Michael Schwameis, Harald Herkner

**Affiliations:** 1 Department of Emergency Medicine, Medical University of Vienna, Vienna, Austria; 2 Department of Clinical Pharmacology, Medical University of Vienna, Vienna, Austria

**Keywords:** blood coagulation, fibrinolysis, immunological models, thromboelastography, tissue plasminogen activator

## Abstract

**Background:**

Biomarkers of fibrinolysis are elevated during acute immunologic reactions (allergic reactions and angioedema), although it is unclear whether fibrinolysis is associated with disease severity.

**Objectives:**

We investigated a possible association between maximum lysis (ML) measured by thromboelastography and the severity of acute immunologic reactions.

**Methods:**

We recruited patients with acute immunologic reactions at a high-volume emergency department. Clinical disease severity at presentation and at the end of the emergency department stay was assessed using a 5-grade scale, ranging from local symptoms to cardiac arrest. We determined ML on admission by thromboelastography (ROTEM's extrinsic [EXTEM], and aprotinin [APTEM] tests), expressed as ML%. Hyperfibrinolysis was defined as an ML of >15% in EXTEM, which was reversed by adding aprotinin (APTEM). We used exact logistic regression to investigate an association between ML% and disease severity (grades 1 and 2 [mild] vs 3-5 [severe]) and between hyperfibrinolysis and disease severity.

**Results:**

We included 31 patients (71% female; median age, 52 [IQR, 35-58] years; 10 [32%] with a severe reaction). ML% was higher in patients with severe symptoms (21 [IQR, 12-100] vs 10 [IQR, 4-17]). Logistic regression found a significant association between ML% and symptom severity (odds ratio, 1.07; 95% CI, 1.01-1.21; *P* = .003). Hyperfibrinolysis was detected in 6 patients and found to be associated with severe symptoms (odds ratio, 17.59; 95% CI, 1.52-991.09; *P* = .02). D-dimer, tryptase, and immunoglobulin E concentrations increased with the severity of immunologic reactions.

**Conclusion:**

ML, quantified by thromboelastography, is associated with the severity of acute immunologic reactions.

## Essentials

• Acute immunologic reactions can lead to alterations in blood clotting.
• We investigated these alterations in emergency department patients using thromboelastography.
• The blood clots of patients with more severe immunologic reactions seem to be more unstable.
• Thromboelastography might be helpful to identify patients who need to be monitored closely.

## 1 Introduction

Acute immunologic reactions include allergic reactions and angioedema. The underlying mechanism for allergic reactions is mast cell degranulation mediated by immunoglobulin E (IgE), leading to histamine release [1]. Angioedema, on the other hand, is caused by a defective protease inhibitor (C1 inhibitor) that causes elevated levels of histamine and/or bradykinin [2]. The clinical distinction between angioedema and allergic reactions is often impossible [3]. The World Allergy Organization (WAO) distinguishes 5 grades of reactions, ranging from mild localized symptoms to cardiovascular collapse (Supplementary Table S1) [4]. Grades 3 to 5 are defined as anaphylaxis [4]. The assessment of the clinical severity of immunologic reactions is difficult because symptoms may have a delayed onset [5]. Current guidelines recommend that even individuals with mild symptoms should be observed for at least 6 to 12 hours [5]. Furthermore, concomitant conditions, especially cardiovascular or respiratory diseases, may influence hemodynamic stability and respiratory symptoms, rendering a rapid assessment of the severity of acute immunologic reactions almost impossible [3,5].

Blood parameters are hardly helpful in the early assessment of patients. Tryptase, histamine, and total or specific IgE concentrations are all well-established parameters for the assessment of immunologic reactions, but they are not ubiquitously available and long turnaround times preclude their use in the acute care setting [4,6]. Thus, novel parameters that allow a quick and reliable assessment of the severity of immunologic reactions are desperately needed.

In a murine model of anaphylaxis, the contact activation system, especially the activation of factor (F)XII, was demonstrated to be a critical regulator of the severity of immunologic reactions [7]. The drop in blood pressure during induced anaphylaxis was much less pronounced in FXII knockout mice, while reconstitution with human FXII induced severe hemodynamic instability [7]. Furthermore, in patients with anaphylaxis, the consumption of FXII was shown [7]. Although not fully elucidated, the current hypothesis involves the release of proinflammatory mediators of the proteases tryptase and chymase and of the negatively charged proteoglycan heparin from activated mast cells [6,8]. Negatively charged molecules activate FXII, which triggers the intrinsic coagulation cascade as well as results in the cleavage of prekallikrein from kallikrein and the formation of bradykinin from high-molecular-weight kininogen [6,8]. In this context, the similarities with hereditary angioedema become apparent: the lack or dysfunction of the serine protease inhibitor C1 inhibitor leads to a failure to inhibit FXIIa and kallikrein (among other proteases), ultimately causing activation of coagulation and bradykinin formation [9]. Furthermore, van den Linden et al. [10] reported the release of tissue-type plasminogen activator (tPA) and von Willebrand factor during anaphylactic reactions caused by insect venom. Of note, prekallikrein, FXIIa, and plasmin may activate urokinase plasminogen activator, while tPA, which is synthesized in endothelial cells, may be released upon stimulation with thrombin, histamine, and bradykinin, among others [[11], [12], [13]]. Additionally, vasoconstrictors frequently used in treating anaphylactic reactions may cause the liberation of protein S and tPA [14]. Interestingly, several case reports describe hyperfibrinolysis in patients suffering from anaphylaxis and increased D-dimer in individuals with chronic urticaria [[15], [16], [17]].

Thromboelastography is a point-of-care test readily available in many acute care settings worldwide [18]. It investigates the viscoelastic properties of whole blood in the presence or absence of specific stimulators of coagulation [19]. Thromboelastography is easy to use and rapidly provides available information about clot formation, stabilization, and degradation [19].

Thromboelastography is also frequently used to detect (hyper)fibrinolysis, although its sensitivity to detect moderate or mild forms is limited [16].

### 1.1 Aim

We aimed to investigate whether maximum lysis (ML) measured by thromboelastography is related to disease severity and progression in patients with acute immunologic reactions. We also describe the relationship between clinical presentation, serum immunologic parameters, and thromboelastography results.

## 2 Methods

Patients were recruited at the emergency department of an academic tertiary care hospital. All adults (>18 years of age) with a suspected allergic reaction or angioedema between July 2021 and April 2023 were eligible to participate in the study. We examined the immunologic history of the patients to identify possible triggers. The severity of the immunologic reaction was assessed at the patient’s presentation and the end of the emergency department stay (follow-up), ie, discharge or transfer. On admission, we performed an extrinsic (EXTEM) test using citrated whole blood with a ROTEM Delta device (Werfen GmbH). In the EXTEM assay, tissue factor was used to activate coagulation. The main parameter of interest was ML (%). An ML of >15% was defined as increased fibrinolysis, in line with other studies [20,21]. Furthermore, we performed an aprotinin (APTEM) assay, a modified EXTEM assay with additional aprotinin. Aprotinin inhibits tPA and should thereby reverse increased fibrinolysis. We defined hyperfibrinolysis as an increased fibrinolysis (ML > 15%), which was reversible by the addition of aprotinin. We also collected blood samples to measure D-dimer concentrations (measured in fibrinogen equivalent units by an immunoturbidimetric assay using latex agglutination [STA Liatest D-Di PLUS, Diagnostica Stago S.A.S.]) and the immunologic parameters histamine, tryptase, and IgE as well as C-reactive protein via our hospital’s central laboratory.

The severity of the immunologic reaction was graded using a simplified version of the WAO classification system (Table 1) [4,22]. Severity staging was performed by clinicians of the research team. All case report forms together with the patient records were reviewed by the same clinicians to ensure consistency.

**Table 1:** Simplified World Allergy Organization system for the grading of the severity of acute immunologic reactions (adapted from [4]).

WAO grading	Severity	Clinical symptoms
Grade 1	Local reaction only	Focal swelling or redness of skin or mucosa
Grade 2	Light systemic reaction	Flush, pruritus, urticaria, rhinorrhea, dyspnea, restlessness, headaches, hoarseness
Grade 3	Severe systemic reaction	Dyspnea, bronchospasm, tachycardia, hypotension, laryngeal edema, nausea, urge to defecate
Grade 4	Life-threatening systemic reaction	Shock, severe dyspnea, bronchospasm, altered mental status, nausea, vomiting, loss of stool or urine
Grade 5	Cardiac arrest	Cardiac arrest, death

WAO, World Allergy Organization.

### 2.1 Statistical analysis

We report the extent of fibrinolysis as ML in percentage in the EXTEM test and baseline characteristics of study patients as total numbers and relative frequencies or medians and IQRs, as appropriate.

In the primary analysis, we compared the extent of ML and immunologic parameters between patients with mild (grades 1 and 2) and severe (grades 3-5) symptoms at admission using exact logistic regression. In addition, we performed ordered logistic regression using the grade of the immunologic reactions at presentation as the dependent variable and ML and patient age as independent variables. We conducted a sensitivity analysis with the same variables using linear regression.

In a secondary analysis, we used exact logistic regression to compare patients who were symptom-free at follow-up and patients who were still symptomatic regarding the extent of ML in EXTEM and immunologic parameters.

To check whether fibrinolysis in general explained our EXTEM results, we also compared ML in APTEM between patients with severe and persistent symptoms and those without these symptoms using exact logistic regression.

In a post-hoc analysis, we investigated an association of hyperfibrinolysis with the severity of acute immunologic reactions. An exact logistic regression model was calculated using the severity of the acute immunologic reaction (mild vs severe) as the dependent variable and the presence of hyperfibrinolysis as the independent variable.

Observations with missing values were excluded from the respective regression models. A 2-sided *P* value of <.05 was considered statistically significant. We used MS Excel 16.75 for data curation. Calculations were performed using Stata SE 17.0 (StataCorp LLC).

The local institutional ethics committee approved the study protocol (vote #1696/2020). The study was conducted in accordance with the principles of the Declaration of Helsinki. All conscious patients gave written informed consent before participating in the study. Unconscious patients were asked for consent after regaining full consciousness. The Strengthening the Reporting of Observational Studies in Epidemiology statement for this manuscript can be found in Supplementary Table S2 [23].

### 2.2 Sample size considerations

A formal sample size calculation was impossible due to the lack of published data. We, therefore, aimed to include all patients with immunologic reactions presenting to our department during the observation period. However, because our hospital focused on treating patients with COVID-19, the number of individuals included in our study was smaller than expected. Following the rule of thumb that at least 10 observations are needed for every independent variable in a regression model, we expected our analyses to reach sufficient power [24].

## 3 Results

**Supplementary Figure S1:**
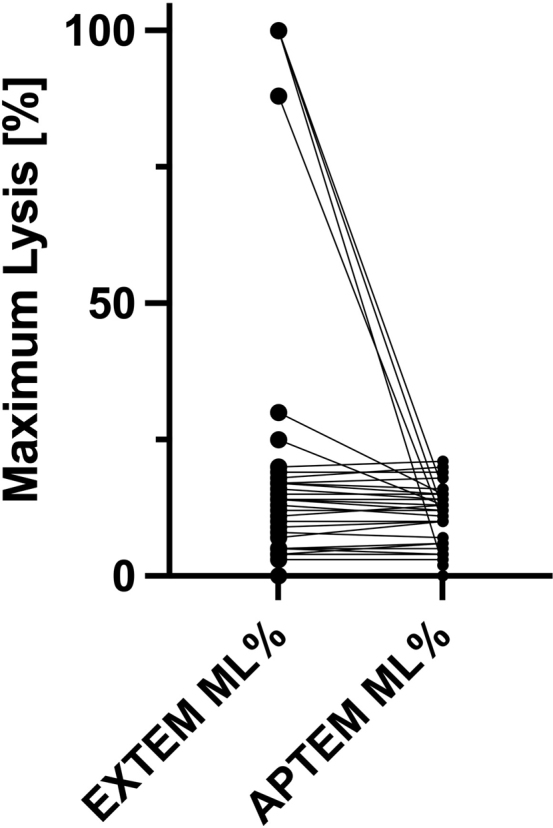
Maximum lysis (ML) before (EXTEM) and after (APTEM) inhibition of fibrinolysis using aprotinin.

**Supplementary Figure S2:**
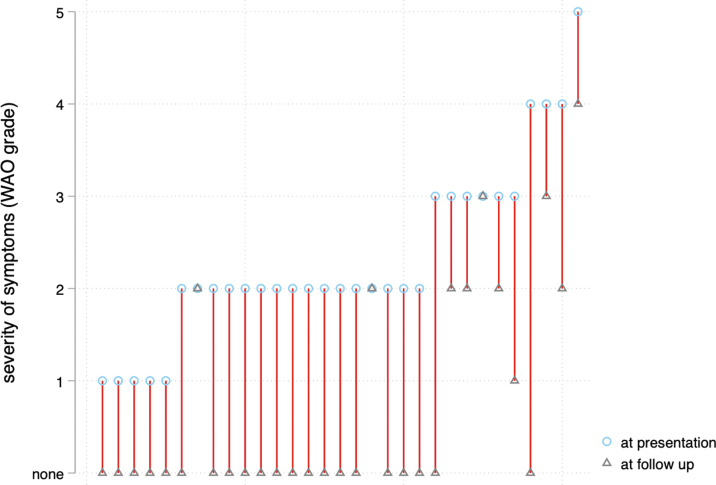
Clinical disease courses of individual patients from admission to the emergency department to follow-up using a simplified World Allergy Organization (WAO) grading system.

We included 31 patients (22 [71%] female; median age, 52 [IQR, 35-58] years). There were no cases of loss to follow-up. The median initial WAO score was 2 (IQR, 2-3). Thromboelastography at presentation showed an ML of >15% in 13 (42%) cases. In 6 patients (19%), we identified hyperfibrinolysis (Supplementary Figure S1). Twenty-seven patients (77%) were discharged from the emergency department and 7 (23%) had to be admitted for further observation. Table 2 contains the baseline characteristics of the study population. Detailed information on the severity of symptoms at presentation and follow-up can be found in Supplementary Figure S2.

**Table 2:** Baseline characteristics of the study population.

Characteristics	All patients, *N* = 31 (100%)	Mild symptoms (WAO grades 1 and 2), *n* = 21 (68%)	Severe symptoms (WAO grades 3-5), *n* = 10 (32%)
Female, *n* (%)	22 (71)	18 (86)	4 (40)
Age (y), median (IQR)	52 (35-58)	52 (32-58)	53 (45-56)
Suspected immunologic trigger			
Medication	16 (52)	9 (43)	7 (70)
Insect venom	6 (19)	4 (5)	2 (20)
Food	5 (16)	4 (5)	1 (10)
Idiopathic/unknown	4 (13)	4 (5)	0 (0)
Onset of symptoms to presentation to ED (min), median (IQR)	83 (42-156)	86 (57-127)	39 (16-210)
ED length of stay (min), median (IQR)	203 (126-361)	159 (88-241)	395 (212-1117)

ED, emergency department; WAO, World Allergy Organization.

### 3.1 Tryptase and histamine levels

Serum tryptase and histamine levels were available in 29 (94%) and 19 (61%) patients, respectively. Both higher serum tryptase and histamine levels were significantly associated with more severe (tryptase: odds ratio [OR], 1.08; 95% CI [1.01-1.17]; *P* = .02; histamine: OR, 1.15; 95% CI [1.02-1.43]; *P* = .002), but not with more persistent (tryptase: OR, 1.03; 95% CI [0.97-1.09]; *P* = .32; histamine: OR, 1.03; 95% CI [0.999-1.1]; *P* = .054) symptoms (Table 3).

**Table 3:** Maximum lysis in the extrinsic (EXTEM) test and immunologic parameters with *P* values derived from exact logistic regression models.

	Symptoms at presentation			Symptoms at follow-up		
	Mild (WAO grades 1 and 2), *n* = 21 (68%)	Severe (WAO grades 3-5), *n* = 10 (32%)	*P* value	Asymptomatic *n* = 21 (68%)	Symptomatic *n* = 10 (32%)	*P* value
ML (%), median (IQR)	10 (5-17)	21 (12-100)	.003	10 (5-17)	17 (12-100)	.02
Tryptase (μg/L), median (IQR)	7.4 (4.6-9)	20.2 (9.3-30)	.02	8.1 (4.6-9.7)	13.2 (5.7-23.9)	.32
IgE (kIU/L), median (IQR)	75.5 (35.2-221)	232 (97.2-763)	.12	81.7 (44.8-332)	146.5 (35.1-763)	.38
Histamine (nmol/L), median (IQR)	9.4 (7.7-11.9)	61 (16.2-93)	.002	9.4 (7.7-11.9)	25.6 (16.2-93)	.054
D-dimer (FEU), μg/mL, median (IQR)	0.46 (0.27-1.45)	1.56 (0.69-2.09)	.48	0.61 (0.27-1.45)	1.56 (0.27-2.09)	.50
C-reactive protein (mg/dL), median (IQR)	0.32 (0.05-0.54)	0.2 (0.11-0.27)	.72	0.32 (0.05-0.58)	0.2 (0.11-0.25)	.66
Fibrinogen (mg/dL), median (IQR)	303 (271-360)	344 (297-362)	.36	330 (271-360)	307 (297-354)	.66
aPTT (s), median (IQR)	34 (32-35)	37 (34-40)	.04	35 (33-35)	36 (30-38)	.47

aPTT, activated partial thromboplastin time; FEU, fibrinogen equivalent units; IgE, immunoglobulin E; ML, maximum lysis; WAO, World Allergy Organization.

### 3.2 ML

**Figure 1:**
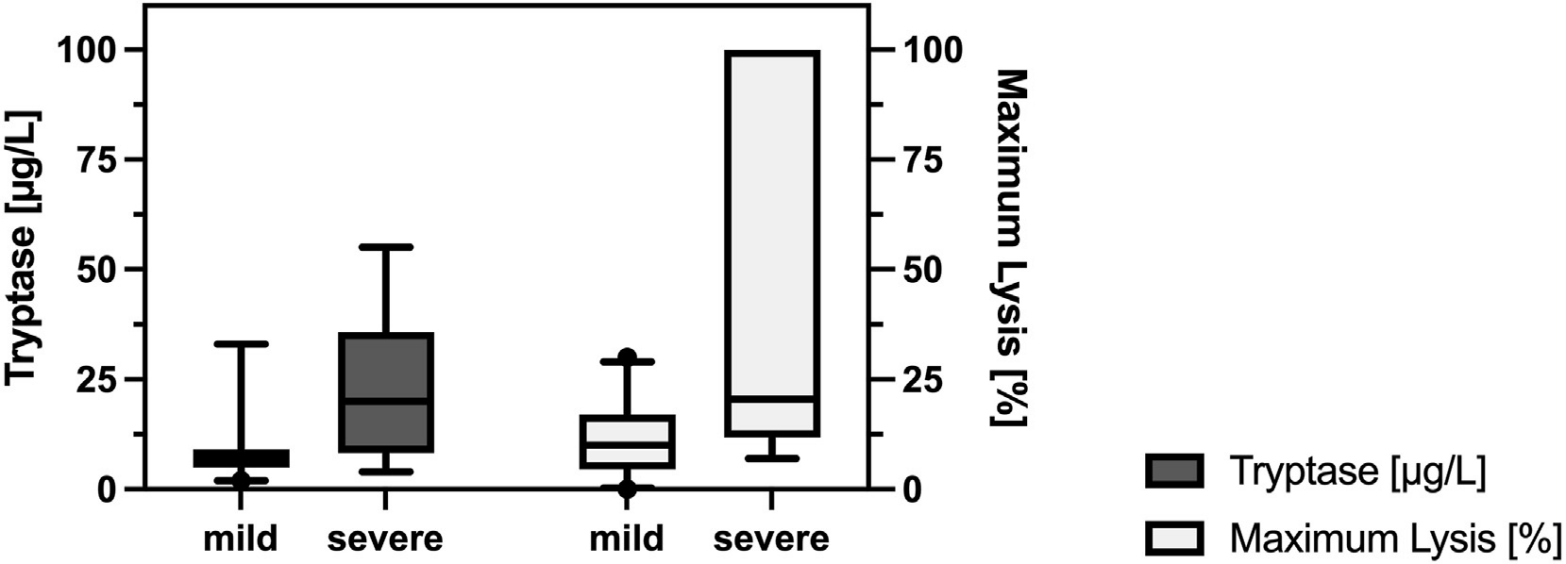
Medians and IQRs for maximum lysis (%) in thromboelastography and serum tryptase levels (μg/L) on admission to the emergency department in mild and severe immunologic reactions

We did not encounter missing data regarding ML in the EXTEM test. The exact logistic regression models showed a significant association of ML between patients with severe symptoms at admission and those without (median, 21% [IQR, 12%-100%] vs 10% [IQR, 4%-17%]; OR, 1.07; 95% CI [1.01-1.21] for every % increase in ML, *P* = .003). Figure 1 shows the distribution of ML and tryptase concentrations in patients with mild and severe symptoms on admission. Furthermore, patients who were still symptomatic at follow-up had higher ML than those who were not (17% [IQR, 12%-100%] vs 10% [IQR, 4%-18%]; OR, 1.03 for every % increase in ML, 95% CI [1.00-1.1]; *P* = .02).

**Figure 2:**
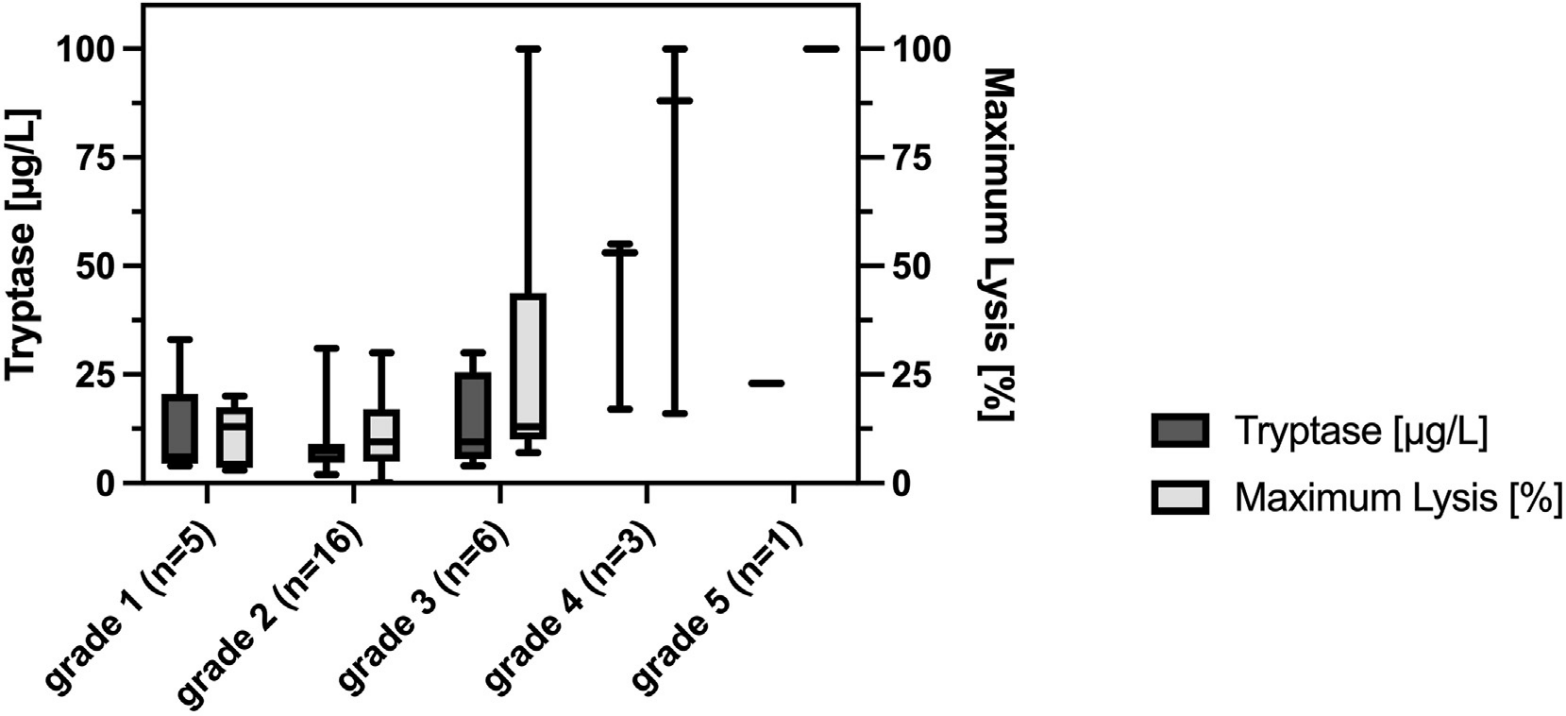
Medians and IQRs for maximum lysis (%) in thromboelastography (EXTEM test) and serum tryptase levels (μg/L) on admission to the emergency department in relation to the severity grades of immunologic reactions. Numbers in parentheses indicate the number of patients in each group.

Ordered logistic regression showed that more severe symptoms at admission were associated with higher amounts of ML (coefficient, .054; 95% CI [0.02-0.09]; *P* = .001). These findings were confirmed by the linear regression model (coefficient, .022; 95% CI [0.01-0.03]; *P* < .001). The results from the ordered logistic regression model did not substantially change after the exclusion of 2 patients (1 with dabigatran and 1 with rivaroxaban) receiving direct oral anticoagulants (coefficient, .051; 95% [0.02-0.08]; *P* = .002). The regression models derived from the primary analyses of EXTEM data can be found in Supplementary Table S3. Figure 2 shows the relation between ML and the severity grades of immunologic reactions on admission.

After aprotinin addition (APTEM assay), ML did not differ significantly between patients with severe and persistent symptoms and those without (median, 13% [IQR, 10%-14%] vs 10% [IQR, 5%-16%]; *P* = .78 and 13% [IQR, 12%-15%] vs 10% [IQR, 5%-15%]; *P* = .37, respectively).

Hyperfibrinolysis was associated with the severity of the acute immunologic reaction with an OR of 17.59 (95% CI [1.52-991.09]; *P* = .02). Supplementary Figure S1 presents ML for each patient before (EXTEM) and after (APTEM) inhibition of fibrinolysis with aprotinin.

Supplementary Table S4 details the predictors of disease severity. Data on the intrinsic coagulation pathway can be found in Supplementary Table S5.

## 4 Discussion

Our results suggest an association between the severity of an acute immunologic reaction and ML measured by thromboelastography. These findings are in accordance with the results of the study by van den Linden et al. [10], who showed increased tPA concentrations and plasmin-antiplasmin complexes in patients with anaphylactic shock. Given its wide availability and rapidly delivered results, thromboelastography may add prognostic information to the current armamentarium of diagnostic means regarding acute immunologic reactions.

The current understanding of the pathophysiologic mechanisms in both hereditary angioedema and anaphylaxis centrally involves the activation of FXII and the consequential activation of coagulation, fibrinolysis, and the formation of bradykinin, which mediates vasodilation, increased vascular permeability, and edema and activates endothelial cells [7,9].

In our patients, the degree of ML was associated with the severity of acute immunologic reactions. Accordingly, D-dimer, a fibrin degradation product, also increased with disease severity, although absolute concentrations remained relatively low, which is in accordance with the findings of the study by van den Linden et al. [10]. However, it is important to note that our population was more heterogeneous as we included all patients with immunologic reactions, regardless of the trigger. In contrast, van den Linden et al. [10] only included individuals allergic to insect venom. In this context, it is essential to differentiate fibrinolysis from fibrinogenolysis, which is tryptase-mediated and may also occur during anaphylaxis [25]. Unlike thrombin, tryptase engages a different fibrinogen cleavage site that prevents clotting [26]. In our population, clotting assays were unaffected, and D-dimer concentrations increased with disease severity.

Upon activation, mast cells release heparin, which may inhibit coagulation [6,8]. While EXTEM is recommended to detect hyperfibrinolysis, this assay has obvious limitations regarding mild-to-moderate effects on clot initiation. We did not observe any dramatic effects of immunologic reactions on activated partial thromboplastin time in our cohort, presumably because the amounts of released endogenous heparin were too low, and routine tests may lack sensitivity in that regard. However, we did not quantify heparin concentrations. Also, tPA, which may also prolong the activated partial thromboplastin time [20], has a short half-life, and any longer delays between the collection and analysis of blood samples may limit the sensitivity of the assay. Furthermore, the sample size of severely affected patients was limited in our cohort. We may not have been able to detect effects on global coagulation assays given the following: (i) the assays’ sensitivity, especially in mild-to-moderate cases, (ii) the relevant interpatient variability, even in severe cases, and (iii) the lack of more specific laboratory assays [7,27].

One patient (a 51-year-old man with no known coronary artery disease) from our population suffered from cardiac arrest. Interestingly, this individual’s serum tryptase level was lower than expected, given the levels of the other patients. This may be because blood sampling in this individual occurred very early (16 minutes) after the onset of symptoms when tryptase levels had not yet peaked [4]. Furthermore, patient-specific characteristics may have contributed to the occurrence of cardiac arrest besides the severity of the anaphylactic reaction. Interestingly, most of the patients from our study are women. Although the reason for this circumstance remains unclear, prior research suggests that female biological sex is a risk factor for immunologic diseases in adults [28].

Thromboelastography might provide valuable clues to identify patients who will need to be monitored longer and more closely at the time of their presentation to the emergency department. This might help to plan and optimize resource allocation. Another aspect could be the modification of therapy. It is still unclear whether fibrinolysis is merely an epiphenomenon of an immunologic response or whether it actively contributes to its severity. After all, it is known that tPA and plasmin disrupt the cerebral endothelial barrier function by bradykinin-dependent mechanisms [[29], [30], [31]]. Therefore, administration of tranexamic acid to counteract hyperfibrinolysis or the bradykinin receptor inhibitor icatibant might be therapeutic approaches worth considering [32]. With our findings as a starting point, future research in a larger patient population will be needed to determine the role of thromboelastography in the diagnosis and treatment of patients with acute immunologic reactions.

A major limitation of this work is the use of only 1 assay to quantify fibrinolysis. We chose to perform thromboelastography because of its wide availability and simplicity of use and because it is a point-of-care assay, which may be performed within the emergency department, delivering data on clot formation within a few minutes and information about (hyper)fibrinolysis within 1 to 2 hours [19,21]. In this context, Raza et al. [33] demonstrated that the sensitivity of thromboelastography is limited in mild and moderate cases of (hyper)fibrinolysis. Thus, we may have overlooked mild and moderate cases of (hyper)fibrinolysis. Other assays may be more apt to provide specific information on the different contributors of fibrinolysis. For instance, enzyme kinetics may be quantified in purified systems using chromogenic or fluorogenic substrates. However, they have limitations with regard to neglecting interactions with other regulatory proteins that are important *in vivo*. Furthermore, such assays are labor intensive, and there is some interlaboratory variability based on the exact composition of the system [34]. Some assays are based on euglobulin. It is important to note that in the preparation process of euglobulin, large amounts of alpha-2-antiplasmin, plasminogen activator inhibitor-1 (PAI-1), and thrombin activatable fibrinolysis inhibitor (TAFI) are lost, impacting the balance of pro- and hypofibrinolytic agents. The measurement of euglobulin lysis time is thereby largely dependent on remaining PAI-1 concentrations and free tPA concentrations and is affected by the method of euglobulin preparation [34]. The quantification of biomarkers of fibrinolysis is limited by the short half-life of critical regulators of fibrinolysis, such as tPA [35]. To reduce relevant preanalytic variability, blood samples need to be processed almost immediately, and a high degree of standardization is required, which was beyond the feasibility of this study, given that immunologic reactions are rare and that this would have required a permanent availability of study staff. That being said, concentrations of D-dimer, a fibrin degradation product and a robust marker of fibrinolysis [10], were quantified. Kuiper et al. [36] spiked blood samples with various concentrations of recombinant tPA and performed thromboelastometry. This assay has shown interesting results for the detection of hypofibrinolytic states during sepsis or after surgeries [36]. It is possible that the addition of low concentrations of tPA may improve the sensitivity of thromboelastography for detecting mild and moderate cases of fibrinolysis. Raza et al. [33] also demonstrated that in severe cases of hyperfibrinolysis, there is a divergence of pro- and antifibrinolytic substances, which may be a requirement for thromboelastography to detect increased fibrinolysis. Additions of small concentrations of tPA may help to tip the balance toward fibrinolysis and improve its sensitivity. However, the optimal concentration of tPA is yet to be determined, which was not within the scope of this study.

Our study has several other limitations. First, our sample size is relatively small. Second, we did not distinguish between allergic reactions and possible angioedema. Third, we neither collected data regarding the sociocultural background nor regarding the ethnic background of our patients. It remains unclear whether these aspects affect fibrinolysis. There are only few data available on ethnical differences in fibrinolysis that suggest only marginal differences unlikely to affect the study outcome, especially given the large effect sizes observed in this study [37]. Finally, in other immunologic responses, such as severe infections or sepsis, tPA may be released and cause an increased ML in thromboelastography [38]. However, C-reactive protein levels were low in our patients.

## 5 Conclusions

More severe and persisting acute immunologic reactions are associated with higher ML in thromboelastography. Whether the latter is a valuable tool for the early identification of patients with unfavorable disease courses could be a subject of future research.
