# Supplementary material for: Thromboelastography in acute immunologic reactions: a prospective pilot study
Source: Res Pract Thromb Haemost. 2024 Apr 27;8(4):102425. doi: 10.1016/j.rpth.2024.102425 (PMC11225642; doi:10.1016/j.rpth.2024.102425)
Supplement: Supplemental 1 [file mmc1.docx]

Supplementary Table S1. Summary of the World Allergy Organization (WAO) system for the grading of the severity of acute immunologic reactions. (adapted from [4])

| WAO Grading | Symptoms |
| --- | --- |
| Grade 1 | Symptoms from 1 organ system |
| Grade 2 | Symptoms from 2 organ systems |
| Grade 3 | Mild lower airway symptoms and/or abdominal symptoms and symptoms from grade 1 |
| Grade 4 | Severe lower airway and/or abdominal symptoms and symptoms from grades 1 or 3 |
| Grade 5 | Respiratory failure and/or cardiovascular collapse and/or loss of consciousness and symptoms from grades 1, 3, or 4 |
