# Supplementary material for: Thromboelastography in acute immunologic reactions: a prospective pilot study
Source: Res Pract Thromb Haemost. 2024 Apr 27;8(4):102425. doi: 10.1016/j.rpth.2024.102425 (PMC11225642; doi:10.1016/j.rpth.2024.102425)
Supplement: Supplemental 3 [file mmc3.docx]

Supplementary Table S3. Regression Models.

## Exact logistic regression models

**Exact logistic regression**

| severe symptoms | Odds Ratio | p-value | [95% Conf | Interval] | Sig |
| --- | --- | --- | --- | --- | --- |
| maximum lysis (%) | 1.07 | .003 | 1.014 | 1.206 | *** |

*____________________________________________________________________*

**** p<.01, ** p<.05, * p<.1*

**Exact logistic regression**

| severe symptoms | Odds Ratio | p-value | [95% Conf | Interval] | Sig |
| --- | --- | --- | --- | --- | --- |
| maximum lysis (%) | 1.051 | .07 | .998 | 1.207 | * |
| tryptase (µg/L) | 1.047 | .29 | .967 | 1.147 |  |

*____________________________________________________________________*

**** p<.01, ** p<.05, * p<.1*

**Exact logistic regression**

| severe symptoms | Odds Ratio | p-value | [95% Conf | Interval] | Sig |
| --- | --- | --- | --- | --- | --- |
| maximum lysis (%) | 1.094 | .015 | 1.007 | 1.842 | ** |
| IgE (kIU/L) | 1.002 | .291 | .999 | 1.005 |  |

*____________________________________________________________________*

**** p<.01, ** p<.05, * p<.1*

**Exact logistic regression**

| severe symptoms | Odds Ratio | p-value | [95% Conf | Interval] | Sig |
| --- | --- | --- | --- | --- | --- |
| maximum lysis (%) | 1 |  | 0 | +infinity |  |
| histamine (nmol/L) | 1.239 | .021 | 1.008 | +infinity |  |

*____________________________________________________________________*

**** p<.01, ** p<.05, * p<.1*

**Exact logistic regression**

| severe symptoms | Odds Ratio | p-value | [95% Conf | Interval] | Sig |
| --- | --- | --- | --- | --- | --- |
| maximum lysis (%) | 1.238 | .003 | .02 | +infinity | *** |
| D-dimer (µg/mL) | .995 | .774 | .658 | 1.425 |  |

*____________________________________________________________________*

**** p<.01, ** p<.05, * p<.1*

**Exact logistic regression**

| severe symptoms | Odds Ratio | p-value | [95% Conf | Interval] | Sig |
| --- | --- | --- | --- | --- | --- |
| maximum lysis (%) | 1.111 | .001 | 1.017 | 1.555 | *** |
| C-reactive protein (mg/dL) | 1.076 | .556 | .757 | 1.555 |  |

*____________________________________________________________________*

**** p<.01, ** p<.05, * p<.1*

**Exact logistic regression**

| severe symptoms | Odds Ratio | p-value | [95% Conf | Interval] | Sig |
| --- | --- | --- | --- | --- | --- |
| maximum lysis (%) | 1.075 | .001 | 1.017 | 1.228 | *** |
| fibrinogen (mg/dL) | 1.009 | .118 | .998 | 1.02 |  |

*____________________________________________________________________*

**** p<.01, ** p<.05, * p<.1*

**Exact logistic regression**

| severe symptoms | Odds Ratio | p-value | [95% Conf | Interval] | Sig |
| --- | --- | --- | --- | --- | --- |
| maximum lysis (%) | 1.063 | .02 | 1.006 | 1.201 | ** |
| aPTT (s) | 1.178 | .324 | .854 | 1.668 |  |

*____________________________________________________________________*

**** p<.01, ** p<.05, * p<.1*

**Exact logistic regression**

| severe symptoms | Odds Ratio | p-value | [95% Conf | Interval] | Sig |
| --- | --- | --- | --- | --- | --- |
| maximum lysis (%) | 1.056 | .036 | 1.002 | 1.23 | ** |
| Lactate (mmol/L) | 2.225 | .113 | .807 | 9.181 |  |

*____________________________________________________________________*

**** p<.01, ** p<.05, * p<.1*

**Exact logistic regression**

| severe symptoms | Odds Ratio | p-value | [95% Conf | Interval] | Sig |
| --- | --- | --- | --- | --- | --- |
| maximum lysis (%) | 1.088 | .001 | 1.018 | 1.241 | *** |
| age (years) | 1.048 | .169 | .984 | 1.136 |  |

*____________________________________________________________________*

**** p<.01, ** p<.05, * p<.1*

**Exact logistic regression**

| severe symptoms | Odds Ratio | p-value | [95% Conf | Interval] | Sig |
| --- | --- | --- | --- | --- | --- |
| maximum lysis (%) | 1.07 | .005 | 1.013 | 1.21 | *** |
| female | .134 | .114 | .003 | 1.411 |  |

*____________________________________________________________________*

**** p<.01, ** p<.05, * p<.1*

**Exact logistic regression**

| severe symptoms | Odds Ratio | p-value | [95% Conf | Interval] | Sig |
| --- | --- | --- | --- | --- | --- |
| maximum lysis (%) | 1.07 | .005 | 1.012 | 1.224 | *** |
| clotting time (s) | 1.008 | .83 | .926 | 1.097 |  |

*____________________________________________________________________*

**** p<.01, ** p<.05, * p<.1*

**Exact logistic regression**

| severe symptoms | Odds Ratio | p-value | [95% Conf | Interval] | Sig |
| --- | --- | --- | --- | --- | --- |
| maximum lysis (%) | 1.061 | .004 | 1.012 | 1.18 | *** |
| clot formation time (s) | .978 | .387 | .926 | 1.022 |  |

*____________________________________________________________________*

**** p<.01, ** p<.05, * p<.1*

**Exact logistic regression**

| Symptoms at follow-up | Odds Ratio | p-value | [95% Conf | Interval] | Sig |
| --- | --- | --- | --- | --- | --- |
| maximum lysis (%) | 1.03 | .021 | 1.004 | 1.072 | ** |

*____________________________________________________________________*

**** p<.01, ** p<.05, * p<.1*

**Exact logistic regression**

| Symptoms at follow-up | Odds Ratio | p-value | [95% Conf | Interval] | Sig |
| --- | --- | --- | --- | --- | --- |
| tryptase (µg/L) | 1.027 | .321 | .974 | 1.088 |  |

*____________________________________________________________________*

**** p<.01, ** p<.05, * p<.1*

**Exact logistic regression**

| Symptoms at follow-up | Odds Ratio | p-value | [95% Conf | Interval] | Sig |
| --- | --- | --- | --- | --- | --- |
| IgE (kIU/L) | 1.001 | .378 | .999 | 1.03 |  |

*____________________________________________________________________*

**** p<.01, ** p<.05, * p<.1*

**Exact logistic regression**

| Symptoms at follow-up | Odds Ratio | p-value | [95% Conf | Interval] | Sig |
| --- | --- | --- | --- | --- | --- |
| histamine (nmol/L) | 1.032 | .054 | .999 | 1.08 | * |

*____________________________________________________________________*

**** p<.01, ** p<.05, * p<.1*

**Exact logistic regression**

| Symptoms at follow-up | Odds Ratio | p-value | [95% Conf | Interval] | Sig |
| --- | --- | --- | --- | --- | --- |
| maximum lysis (%) | 1.029 | .033 | 1.002 | 1.077 | ** |
| D-dimer (µg/mL) | 1.032 | .731 | .784 | 1.417 |  |

*____________________________________________________________________*

**** p<.01, ** p<.05, * p<.1*

**Exact logistic regression**

| Symptoms at follow-up | Odds Ratio | p-value | [95% Conf | Interval] | Sig |
| --- | --- | --- | --- | --- | --- |
| C-reactive protein (mg/dL) | .784 | .657 | .276 | 1.329 |  |

*____________________________________________________________________*

**** p<.01, ** p<.05, * p<.1*

**Exact logistic regression**

| Symptoms at follow-up | Odds Ratio | p-value | [95% Conf | Interval] | Sig |
| --- | --- | --- | --- | --- | --- |
| fibrinogen (mg/dL) | .998 | .661 | .988 | 1.007 |  |

*____________________________________________________________________*

**** p<.01, ** p<.05, * p<.1*

**Exact logistic regression**

| Symptoms at follow-up | Odds Ratio | p-value | [95% Conf | Interval] | Sig |
| --- | --- | --- | --- | --- | --- |
| aPTT (s) | 1.075 | .472 | .883 | 1.316 |  |

*____________________________________________________________________*

**** p<.01, ** p<.05, * p<.1*

**Exact logistic regression**

| Symptoms at follow-up | Odds Ratio | p-value | [95% Conf | Interval] | Sig |
| --- | --- | --- | --- | --- | --- |
| lactate (mmol/L) | 1.393 | .313 | .736 | 2.784 |  |

*____________________________________________________________________*

**** p<.01, ** p<.05, * p<.1*

**Exact logistic regression**

| Symptoms at follow-up | Odds Ratio | p-value | [95% Conf | Interval] | Sig |
| --- | --- | --- | --- | --- | --- |
| maximum lysis (%) | 1.031 | .017 | 1.005 | 1.075 | ** |
| age (years) | 1.039 | .165 | .985 | 1.104 |  |

*____________________________________________________________________*

**** p<.01, ** p<.05, * p<.1*

**Exact logistic regression**

| Symptoms at follow-up | Odds Ratio | p-value | [95% Conf | Interval] | Sig |
| --- | --- | --- | --- | --- | --- |
| maximum lysis (%) | 1.032 | .03 | 1.003 | 1.08 | ** |
| female | .067 | .018 | .001 | .701 |  |

*____________________________________________________________________*

**** p<.01, ** p<.05, * p<.1*

**Exact logistic regression**

| Symptoms at follow-up | Odds Ratio | p-value | [95% Conf | Interval] | Sig |
| --- | --- | --- | --- | --- | --- |
| clotting time (s) | .999 | 1 | .93 | 1.07 |  |

*____________________________________________________________________*

**** p<.01, ** p<.05, * p<.1*

**Exact logistic regression**

| Symptoms at follow-up | Odds Ratio | p-value | [95% Conf | Interval] | Sig |
| --- | --- | --- | --- | --- | --- |
| clot formation time (s) | 0.988 | .491 | .954 | 1.018 |  |

*____________________________________________________________________*

**** p<.01, ** p<.05, * p<.1*

## Ordered logistic regression models

**Ordered logistic regression**

| symptom severity (stages 1-5) | Coef. | St.Err. | t-value | p-value | [95% Conf | Interval] | Sig |
| --- | --- | --- | --- | --- | --- | --- | --- |
| maximum lysis (%) | .054 | .017 | 3.18 | .001 | .021 | .087 | *** |
| cut1 | -.9 | .539 |  |  | -1.957 | .157 |  |
| cut2 | 1.897 | .558 |  |  | .803 | 2.991 |  |
| cut3  cut4 | 4.018  6.393 | 1.124  1.724 |  |  | 1.815  3.013 | 6.221  9.772 |  |
|  | | | | | | | |
| **** p<.01, ** p<.05, * p<.1* | | | | | | | |

**Ordered logistic regression**

| symptom severity (stages 1-5) | Coef. | St.Err. | t-value | p-value | [95% Conf | Interval] | Sig |
| --- | --- | --- | --- | --- | --- | --- | --- |
| maximum lysis (%) | .044 | .018 | 2.47 | .014 | .009 | .079 | ** |
| tryptase (µg/L) | .042 | .034 | 1.24 | .217 | -.025 | .11 |  |
| cut1 | -.505 | .599 |  |  | -1.679 | .67 |  |
| cut2 | 2.147 | .654 |  |  | .865 | 3.428 |  |
| cut3 | 4.465 | 1.281 |  |  | 1.955 | 6.975 |  |
| cut4 | 6.841 | 1.827 |  |  | 3.259 | 10.423 |  |
|  | | | | | | | |
| **** p<.01, ** p<.05, * p<.1*  **Ordered logistic regression**   \| symptom severity (stages 1-5) \| Coef. \| St.Err. \| t-value \| p-value \| [95% Conf \| Interval] \| Sig \| \| --- \| --- \| --- \| --- \| --- \| --- \| --- \| --- \| \| maximum lysis (%) \| .051 \| .016 \| 3.08 \| .002 \| .018 \| .083 \| *** \| \| IgE (kIU/L) \| .002 \| .001 \| 1.44 \| .150 \| -.001 \| .004 \|  \| \| cut1 \| -.588 \| .569 \|  \|  \| -1.704 \| .528 \|  \| \| cut2 \| 2.249 \| .652 \|  \|  \| .971 \| 3.526 \|  \| \| cut3 \| 4.373 \| 1.134 \|  \|  \| 2.149 \| 6.596 \|  \| \| cut4 \| 7.056 \| 1.907 \|  \|  \| 3.319 \| 10.794 \|  \| \|  \| \| \| \| \| \| \| \| \| **** p<.01, ** p<.05, * p<.1* \| \| \| \| \| \| \| \| | | | | | | | |

**Ordered logistic regression**

| symptom severity (stages 1-5) | Coef. | St.Err. | t-value | p-value | [95% Conf | Interval] | Sig |
| --- | --- | --- | --- | --- | --- | --- | --- |
| maximum lysis (%) | .068 | .052 | 1.31 | .191 | -.034 | .169 |  |
| histamine (nmol/L) | .032 | .021 | 1.57 | .115 | -.008 | .073 |  |
| cut1 | -.915 | .912 |  |  | -2.703 | .873 |  |
| cut2 | 2.462 | .957 |  |  | .586 | 4.338 |  |
| cut3 | 8.746 | 5.069 |  |  | -1.189 | 18.681 |  |
|  | | | | | | | |
| **** p<.01, ** p<.05, * p<.1* | | | | | | | |

**Ordered logistic regression**

| symptom severity (stages 1-5) | Coef. | St.Err. | t-value | p-value | [95% Conf | Interval] | Sig |
| --- | --- | --- | --- | --- | --- | --- | --- |
| maximum lysis (%) | .054 | .017 | 3.09 | .002 | .02 | .088 | *** |
| D-dimer (µg/mL) | .038 | .138 | 0.27 | .784 | -.232 | .308 |  |
| cut1 | -.958 | .621 |  |  | -2.176 | .259 |  |
| cut2 | 2.057 | .629 |  |  | .824 | 3.289 |  |
| cut3 | 4.041 | 1.175 |  |  | 1.739 | 6.344 |  |
| cut4 | 6.54 | 1.864 |  |  | 2.887 | 10.193 |  |
|  | | | | | | | |
| **** p<.01, ** p<.05, * p<.1* | | | | | | | |
| **Ordered logistic regression**   \| symptom severity (stages 1-5) \| Coef. \| St.Err. \| t-value \| p-value \| [95% Conf \| Interval] \| Sig \| \| --- \| --- \| --- \| --- \| --- \| --- \| --- \| --- \| \| maximum lysis (%) \| .056 \| .017 \| 3.25 \| .001 \| .022 \| .09 \| *** \| \| C-reactive protein (mg/dL) \| .112 \| .172 \| 0.65 \| .515 \| -.226 \| .45 \|  \| \| cut1 \| -.742 \| .56 \|  \|  \| -1.838 \| .355 \|  \| \| cut2 \| 2.276 \| .659 \|  \|  \| .985 \| 3.567 \|  \| \| cut3 \| 4.242 \| 1.179 \|  \|  \| 1.932 \| 6.552 \|  \| \| cut4 \| 6.645 \| 1.772 \|  \|  \| 3.172 \| 10.117 \|  \| \|  \| \| \| \| \| \| \| \| \| **** p<.01, ** p<.05, * p<.1* \| \| \| \| \| \| \| \| \|  \| \| \| \| \| \| \| \|   **Ordered logistic regression**   \| symptom severity (stages 1-5) \| Coef. \| St.Err. \| t-value \| p-value \| [95% Conf \| Interval] \| Sig \| \| --- \| --- \| --- \| --- \| --- \| --- \| --- \| --- \| \| maximum lysis (%) \| .063 \| .018 \| 3.53 \| <.001 \| .028 \| .097 \| *** \| \| fibrinogen (mg/dL) \| .009 \| .005 \| 2.06 \| .039 \| 0 \| .018 \| ** \| \| cut1 \| 2.106 \| 1.539 \|  \|  \| -.911 \| 5.124 \|  \| \| cut2 \| 5.177 \| 1.735 \|  \|  \| 1.777 \| 8.577 \|  \| \| cut3 \| 7.542 \| 2.17 \|  \|  \| 3.29 \| 11.795 \|  \| \| cut4 \| 10.246 \| 2.716 \|  \|  \| 4.923 \| 15.569 \|  \| \|  \| \| \| \| \| \| \| \| \| **** p<.01, ** p<.05, * p<.1* \| \| \| \| \| \| \| \|   **Ordered logistic regression**   \| symptom severity (stages 1-5) \| Coef. \| St.Err. \| t-value \| p-value \| [95% Conf \| Interval] \| Sig \| \| --- \| --- \| --- \| --- \| --- \| --- \| --- \| --- \| \| maximum lysis (%) \| .051 \| .018 \| 2.90 \| .004 \| .016 \| .085 \| *** \| \| aPTT (s) \| .1 \| .106 \| 0.95 \| .344 \| -.108 \| .308 \|  \| \| cut1 \| 2.476 \| 3.603 \|  \|  \| -4.586 \| 9.539 \|  \| \| cut2 \| 5.346 \| 3.714 \|  \|  \| -1.933 \| 12.625 \|  \| \| cut3 \| 7.496 \| 3.879 \|  \|  \| -.106 \| 15.098 \|  \| \| cut4 \| 9.914 \| 4.221 \|  \|  \| 1.64 \| 18.187 \|  \| \|  \| \| \| \| \| \| \| \| \| **** p<.01, ** p<.05, * p<.1* \| \| \| \| \| \| \| \| \|  \| \| \| \| \| \| \| \|   **Ordered logistic regression**   \| symptom severity (stages 1-5) \| Coef. \| St.Err. \| t-value \| p-value \| [95% Conf \| Interval] \| Sig \| \| --- \| --- \| --- \| --- \| --- \| --- \| --- \| --- \| \| maximum lysis (%) \| .044 \| .019 \| 2.28 \| .023 \| .006 \| .081 \| ** \| \| lactate (mmol/L) \| .45 \| .406 \| 1.11 \| .267 \| -.345 \| 1.246 \|  \| \| cut1 \| -.267 \| .825 \|  \|  \| -1.884 \| 1.35 \|  \| \| cut2 \| 2.928 \| .976 \|  \|  \| 1.016 \| 4.84 \|  \| \| cut3 \| 4.813 \| 1.464 \|  \|  \| 1.943 \| 7.683 \|  \| \| cut4 \| 7.289 \| 2.067 \|  \|  \| 3.237 \| 11.34 \|  \| \|  \| \| \| \| \| \| \| \| \| **** p<.01, ** p<.05, * p<.1* \| \| \| \| \| \| \| \| | | | | | | | |

**Ordered logistic regression**

| symptom severity (stages 1-5) | Coef. | St.Err. | t-value | p-value | [95% Conf | Interval] | Sig |
| --- | --- | --- | --- | --- | --- | --- | --- |
| maximum lysis (%) | .056 | .018 | 3.17 | .002 | .021 | .09 | *** |
| age (years) | .016 | .022 | 0.73 | .464 | -.027 | .059 |  |
| cut1 | -.108 | 1.196 |  |  | -2.452 | 2.235 |  |
| cut2 | 2.696 | 1.244 |  |  | .258 | 5.133 |  |
| cut3 | 4.891 | 1.676 |  |  | 1.606 | 8.176 |  |
| cut4 | 7.325 | 2.2 |  |  | 3.013 | 11.636 |  |
|  | | | | | | | |
| **** p<.01, ** p<.05, * p<.1* | | | | | | | |
|  | | | | | | | |

**Ordered logistic regression**

| symptom severity (stages 1-5) | Coef. | St.Err. | t-value | p-value | [95% Conf | Interval] | Sig |
| --- | --- | --- | --- | --- | --- | --- | --- |
| maximum lysis (%) | .055 | .017 | 3.17 | .002 | .021 | .089 | *** |
| female | -1.868 | .871 | -2.15 | .032 | -3.575 | -.161 | ** |
| cut1 | -2.435 | .916 |  |  | -4.231 | -.639 |  |
| cut2 | .717 | .754 |  |  | -.761 | 2.195 |  |
| cut3 | 3.018 | 1.165 |  |  | .735 | 5.302 |  |
| cut4 | 5.943 | 1.891 |  |  | 2.238 | 9.649 |  |
|  | | | | | | | |
| **** p<.01, ** p<.05, * p<.1* | | | | | | | |
|  | | | | | | | |

**Ordered logistic regression**

| symptom severity (stages 1-5) | Coef. | St.Err. | t-value | p-value | [95% Conf | Interval] | Sig |
| --- | --- | --- | --- | --- | --- | --- | --- |
| maximum lysis (%) | .052 | .017 | 3.14 | .002 | .02 | .085 | *** |
| clotting time (s) | .024 | .032 | 0.73 | .463 | -.04 | .087 |  |
| cut1 | .611 | 2.123 |  |  | -3.551 | 4.772 |  |
| cut2 | 3.46 | 2.225 |  |  | -.9 | 7.82 |  |
| cut3 | 5.593 | 2.461 |  |  | .77 | 10.417 |  |
| cut4 | 8.013 | 2.846 |  |  | 2.435 | 13.59 |  |
|  | | | | | | | |
| **** p<.01, ** p<.05, * p<.1* | | | | | | | |
|  | | | | | | | |

**Ordered logistic regression**

| symptom severity (stages 1-5) | Coef. | St.Err. | t-value | p-value | [95% Conf | Interval] | Sig |
| --- | --- | --- | --- | --- | --- | --- | --- |
| maximum lysis (%) | .055 | .017 | 3.27 | .001 | .022 | .088 | *** |
| clot formation time (s) | -.015 | .014 | -1.05 | .296 | -.043 | .013 |  |
| cut1 | -2.133 | 1.313 |  |  | -4.706 | .44 |  |
| cut2 | .798 | 1.167 |  |  | -1.49 | 3.085 |  |
| cut3 | 2.881 | 1.519 |  |  | -.096 | 5.859 |  |
| cut4 | 5.211 | 2.005 |  |  | 1.281 | 9.141 |  |
|  | | | | | | | |
| **** p<.01, ** p<.05, * p<.1* | | | | | | | |

## Linear regression models

**Linear regression**

| symptom severity (stages 1-5) | Coef. | St.Err. | t-value | p-value | [95% Conf | Interval] | Sig |
| --- | --- | --- | --- | --- | --- | --- | --- |
| maximum lysis (%) | .022 | .004 | 4.95 | <.001 | .013 | .031 | *** |
| Constant | 1.814 | .167 | 10.85 | <.001 | 1.472 | 2.156 | *** |
|  | | | | | | | |
| **** p<.01, ** p<.05, * p<.1* | | | | | | | |

**Linear regression**

| symptom severity (stages 1-5) | Coef. | St.Err. | t-value | p-value | [95% Conf | Interval] | Sig |
| --- | --- | --- | --- | --- | --- | --- | --- |
| maximum lysis (%) | .018 | .005 | 3.45 | .002 | .007 | .029 | *** |
| tryptase (µg/L) | .016 | .012 | 1.42 | .167 | -.007 | .04 |  |
| Constant | 1.669 | .204 | 8.20 | <.001 | 1.251 | 2.088 | *** |
|  | | | | | | | |
| **** p<.01, ** p<.05, * p<.1* | | | | | | | |
| **Linear regression**   \| symptom severity (stages 1-5) \| Coef. \| St.Err. \| t-value \| p-value \| [95% Conf \| Interval] \| Sig \| \| --- \| --- \| --- \| --- \| --- \| --- \| --- \| --- \| \| maximum lysis (%) \| .02 \| .005 \| 4.29 \| <.001 \| .011 \| .03 \| *** \| \| IgE (kIU/L) \| .001 \| 0 \| 1.44 \| .163 \| 0 \| .001 \|  \| \| Constant \| 1.7 \| .187 \| 9.08 \| <.001 \| 1.316 \| 2.084 \| *** \| \|  \| \| \| \| \| \| \| \| \| **** p<.01, ** p<.05, * p<.1* \| \| \| \| \| \| \| \| | | | | | | | |

**Linear regression**

| symptom severity (stages 1-5) | Coef. | St.Err. | t-value | p-value | [95% Conf | Interval] | Sig |
| --- | --- | --- | --- | --- | --- | --- | --- |
| maximum lysis (%) | .011 | .005 | 2.18 | .045 | 0 | .023 | ** |
| histamine (nmol/L) | .009 | .005 | 1.79 | .092 | -.002 | .019 | * |
| Constant | 1.85 | .187 | 9.89 | 0 | 1.454 | 2.247 | *** |
|  | | | | | | | |
| **** p<.01, ** p<.05, * p<.1* | | | | | | | |

**Linear regression**

| symptom severity (stages 1-5) | Coef. | St.Err. | t-value | p-value | [95% Conf | Interval] | Sig |
| --- | --- | --- | --- | --- | --- | --- | --- |
| maximum lysis (%) | .022 | .005 | 4.69 | <.001 | .012 | .031 | *** |
| D-dimer (µg/mL) | .019 | .044 | 0.42 | .676 | -.072 | .11 |  |
| Constant | 1.791 | .186 | 9.61 | <.001 | 1.407 | 2.174 | *** |
|  | | | | | | | |
| **** p<.01, ** p<.05, * p<.1*  **Linear regression**   \| symptom severity (stages 1-5) \| Coef. \| St.Err. \| t-value \| p-value \| [95% Conf \| Interval] \| Sig \| \| --- \| --- \| --- \| --- \| --- \| --- \| --- \| --- \| \| maximum lysis (%) \| .023 \| .005 \| 5.12 \| <.001 \| .014 \| .032 \| *** \| \| C-reactive protein (mg/dL) \| .034 \| .066 \| 0.52 \| .605 \| -.101 \| .17 \|  \| \| Constant \| 1.73 \| .182 \| 9.51 \| <.001 \| 1.357 \| 2.103 \| *** \| \|  \| \| \| \| \| \| \| \| \| **** p<.01, ** p<.05, * p<.1* \| \| \| \| \| \| \| \| | | | | | | | |
| **Linear regression**   \| symptom severity (stages 1-5) \| Coef. \| St.Err. \| t-value \| p-value \| [95% Conf \| Interval] \| Sig \| \| --- \| --- \| --- \| --- \| --- \| --- \| --- \| --- \| \| maximum lysis (%) \| .023 \| .004 \| 5.48 \| <.001 \| .015 \| .032 \| *** \| \| fibrinogen (mg/dL) \| .003 \| .001 \| 2.11 \| .044 \| 0 \| .006 \| ** \| \| Constant \| .767 \| .521 \| 1.47 \| .153 \| -.301 \| 1.834 \|  \| \|  \| \| \| \| \| \| \| \| \| **** p<.01, ** p<.05, * p<.1*  **Linear regression**   \| symptom severity (stages 1-5) \| \| Coef. \| \| St.Err. \| \| t-value \| \| p-value \| \| [95% Conf \| \| Interval] \| \| Sig \| \| \| --- \| --- \| --- \| --- \| --- \| --- \| --- \| --- \| --- \| --- \| --- \| --- \| --- \| --- \| --- \| --- \| \| maximum lysis (%) \| \| .021 \| \| .005 \| \| 4.09 \| \| <.001 \| \| .01 \| \| .031 \| \| *** \| \| \| aPTT (s) \| \| .023 \| \| .04 \| \| 0.58 \| \| .566 \| \| -.058 \| \| .104 \| \|  \| \| \| Constant \| \| 1.045 \| \| 1.336 \| \| 0.78 \| \| .441 \| \| -1.691 \| \| 3.781 \| \|  \| \| \|  \| \| \| \| \| \| \| \| \| \| \| \| \| \| \| \| \| **** p<.01, ** p<.05, * p<.1* \| \| \| \| \| \| \| \| \| \| \| \| \| \| \| \|   **Linear regression**   \| symptom severity (stages 1-5) \| \| Coef. \| St.Err. \| t-value \| p-value \| [95% Conf \| Interval] \| Sig \| \| --- \| --- \| --- \| --- \| --- \| --- \| --- \| --- \| --- \| \| maximum lysis (%) \| \| .019 \| .005 \| 3.71 \| .001 \| .009 \| .03 \| *** \| \| lactate (mmol/L) \| \| .17 \| .131 \| 1.29 \| .209 \| -.102 \| .441 \|  \| \| Constant \| \| 1.501 \| .276 \| 5.44 \| 0 \| .93 \| 2.072 \| *** \| \|  \| \| \| \| \| \| \| \| \| \| **** p<.01, ** p<.05, * p<.1* \| \| \| \| \| \| \| \| \| \| \| \| \| \| \| \| \| | | | | | | | |

**Linear regression**

| symptom severity (stages 1-5) | Coef. | St.Err. | t-value | p-value | [95% Conf | Interval] | Sig |
| --- | --- | --- | --- | --- | --- | --- | --- |
| maximum lysis (%) | .023 | .005 | 4.99 | <.001 | .013 | .032 | *** |
| age (years) | .007 | .008 | 0.88 | .388 | -.009 | .023 |  |
| Constant | 1.461 | .436 | 3.35 | .002 | .569 | 2.353 | *** |
|  | | | | | | | |
| **** p<.01, ** p<.05, * p<.1* | | | | | | | |
|  | | | | | | | |

**Linear regression**

| symptom severity (stages 1-5) | Coef. | St.Err. | t-value | p-value | [95% Conf | Interval] | Sig |
| --- | --- | --- | --- | --- | --- | --- | --- |
| maximum lysis (%) | .02 | .004 | 4.83 | <.001 | .012 | .029 | *** |
| female | -.629 | .266 | -2.37 | .025 | -1.173 | -.084 | ** |
| Constant | 2.28 | .251 | 9.09 | <.001 | 1.766 | 2.793 | *** |
|  | | | | | | | |
| **** p<.01, ** p<.05, * p<.1* | | | | | | | |

**Linear regression**

| symptom severity (stages 1-5) | Coef. | St.Err. | t-value | p-value | [95% Conf | Interval] | Sig |
| --- | --- | --- | --- | --- | --- | --- | --- |
| maximum lysis (%) | .022 | .005 | 4.66 | <.001 | .012 | .031 | *** |
| clotting time (s) | .006 | .012 | 0.50 | .619 | -.019 | .032 |  |
| Constant | 1.416 | .809 | 1.75 | .091 | -.24 | 3.072 | * |
|  | | | | | | | |
| **** p<.01, ** p<.05, * p<.1* | | | | | | | |
|  | | | | | | | |

**Linear regression**

| symptom severity (stages 1-5) | Coef. | St.Err. | t-value | p-value | [95% Conf | Interval] | Sig |
| --- | --- | --- | --- | --- | --- | --- | --- |
| maximum lysis (%) | .022 | .005 | 4.92 | <.001 | .013 | .032 | *** |
| clot formation time (s) | -.004 | .005 | -0.80 | .433 | -.015 | .006 |  |
| Constant | 2.14 | .443 | 4.83 | <.001 | 1.233 | 3.047 | *** |
|  | | | | | | | |
| **** p<.01, ** p<.05, * p<.1* | | | | | | | |
