# Supplementary material for: Thromboelastography in acute immunologic reactions: a prospective pilot study
Source: Res Pract Thromb Haemost. 2024 Apr 27;8(4):102425. doi: 10.1016/j.rpth.2024.102425 (PMC11225642; doi:10.1016/j.rpth.2024.102425)
Supplement: Supplemental 4 [file mmc4.docx]

Supplementary Table S4. Parameters assessed as predictors of disease severity at presentation. IQR interquartile range. Maximum lysis (ML) derived from EXTEM test.

|  | Grade 1 (n=5) | Grade 2 (n=16) | Grade 3 (n=6) | Grade 4 (n=3) | Grade 5 (n=1) |
| --- | --- | --- | --- | --- | --- |
| ML, %, median (IQR) | 13 (4 – 15) | 10 (5 – 17) | 13 (11 – 25) | 88 (16 – 100) | 100 (100 – 100) |
| Tryptase, µg/L, median (IQR) | 5.5 (5.3 – 8.1) | 7.6 (4.6 – 9) | 9.5 (5.7 – 23.9) | 53.1 (17.1 – 55.1) | 23.2 (23.2 – 23.2) |
| IgE, kIU/L, median (IQR) | 53.1 (33.9 – 267) | 81 (36.4 – 175) | 146.5 (35.1 – 740) | 296 (97.2 – 763) | 1344 (1344 – 1344) |
| Histamine, nmol/L, median (IQR) | 8.1 (5.4 – 10.8) | 9.4 (8.4 – 12.9) | 77 (16.2 – 93) | 23.3 (23.3 – 23.3) | N/A |
| D-dimer, µg/mL, median (IQR) | 1.35 (0.37 – 7.05) | 0.43 (0.27 – 0.66) | 0.72 (0.27 – 1.56) | 2.09 (0.69 – 2.52) | 12.44 (12.44 – 12.44) |
| C-reactive protein, mg/dL, median (IQR) | 0.07 (0.04 – 0.54) | 0.32 (0.13 – 0.51) | 0.25 (0.04 – 2.54) | 0.2 (0.11 – 0.27) | 0.19 (0.19 – 0.19) |
| Fibrinogen, mg/dL, median (IQR) | 276 (238 – 432) | 305 (275 – 357) | 324 (277 – 354) | 348 (297 – 436) | 362 (362 – 362) |
