# Supplementary material for: Thromboelastography in acute immunologic reactions: a prospective pilot study
Source: Res Pract Thromb Haemost. 2024 Apr 27;8(4):102425. doi: 10.1016/j.rpth.2024.102425 (PMC11225642; doi:10.1016/j.rpth.2024.102425)
Supplement: Supplemental 5 [file mmc5.docx]

Supplementary Table S5. Parameters of clot formation from the intrinsic (INTEM) test with p-values derived from exact logistic regression models. IQR interquartile range.

|  | Symptoms at presentation | | | Symptoms at follow-up | | |
| --- | --- | --- | --- | --- | --- | --- |
|  | Mild (grades 1-2) | Severe (grades 3-5) | p-value | Asymptomatic | Symptomatic | p-value |
| Clotting time, s, median (IQR) | 173 (149 – 182) | 164 (120 – 166) | 0.106 | 173 (154 – 182) | 146 (120 – 166) | 0.056 |
| Clot formation time, s, median (IQR) | 61 (48 – 81) | 58 (50 – 96) | 0.839 | 63 (53 – 82) | 55 (49 – 77) | 0.494 |
